# A Robust Method Based on Deep Learning for Compressive Spectrum Sensing

**DOI:** 10.3390/s25072187

**Published:** 2025-03-30

**Authors:** Haoye Zeng, Yantao Yu, Guojin Liu, Yucheng Wu

**Affiliations:** School of Microelectronics and Communication Engineering, Chongqing University, Chongqing 400044, China; zenghaoye@cqu.edu.cn (H.Z.); liuguojin@cqu.edu.cn (G.L.); wuyucheng@cqu.edu.cn (Y.W.)

**Keywords:** compressive spectrum sensing, deep learning, wideband spectrum signal reconstruction, wideband spectrum sensing, block sparsity

## Abstract

In cognitive radio, compressive spectrum sensing (CSS) is critical for efficient wideband spectrum sensing (WSS). However, traditional reconstruction algorithms exhibit suboptimal performance, and conventional WSS methods fail to fully capture the inherent structural information of wideband spectrum signals. Moreover, most existing deep learning-based approaches fail to effectively exploit the sparse structures of wideband spectrum signals, resulting in limited reconstruction performance. To overcome these limitations, we propose BEISTA-Net, a deep learning-based framework for reconstructing compressed wideband signals. BEISTA-Net integrates the iterative shrinkage-thresholding algorithm (ISTA) with deep learning, thereby extracting and enhancing the block sparsity features of wideband spectrum signals, which significantly improves reconstruction accuracy. Next, we propose BSWSS-Net, a lightweight network that efficiently leverages the sparse features of the reconstructed signal to enhance WSS performance. By jointly employing BEISTA-Net and BSWSS-Net, the challenges in CSS are effectively addressed. Extensive numerical experiments demonstrate that our proposed CSS method achieves state-of-the-art performance across both low and high signal-to-noise ratio scenarios.

## 1. Introduction

With the advent of fifth-generation (5G) commercialization, global mobile data consumption and the number of connected devices have surged exponentially, intensifying the scarcity of spectrum resources [1]. The traditional static spectrum allocation strategy leads to inefficient utilization and further exacerbates the conflict between spectrum supply and demand. Cognitive radio technology presents a promising solution by dynamically analyzing the spectrum environment and enabling secondary users (SUs) to opportunistically access an underutilized spectrum, known as spectrum holes [2]. As the cornerstone of cognitive radio, spectrum sensing is crucial for ensuring its operational effectiveness. To identify more available channels, wideband spectrum sensing (WSS) has been proposed.

Traditional WSS techniques rely on the Nyquist sampling theorem, which mandates a sampling rate of at least twice the maximum signal frequency. Recently, WSS based on compressed sensing theory has emerged as a promising alternative, significantly reducing the sampling rate requirements for analog-to-digital converters (ADCs). Compressive spectrum sensing (CSS) algorithms can be broadly categorized into two types. The first type avoids signal reconstruction and directly assesses spectrum occupancy from compressive measurements [3]. The second type decomposes CSS into two steps: initially performing sparse signal reconstruction, followed by utilizing the reconstructed signals for WSS [4].

In cognitive radio, compressed sensing (CS) leverages the sparsity of wideband spectrum signals in domains such as frequency and spatial, thereby substantially reducing the sampling rate requirements for devices and enabling cost-effective wideband spectrum sampling [5]. The original signal can be reconstructed from low-rank subsamples using specialized algorithms. Traditional CS reconstruction algorithms can be broadly categorized into two types: greedy algorithms and convex optimization-based algorithms, both of which have achieved significant advancements. Greedy algorithms include methods such as the matching pursuit (MP) algorithm [6], orthogonal matching pursuit (OMP) algorithm [7], regularized orthogonal matching pursuit (ROMP) algorithm [8], and compressive sampling matching pursuit (CoSaMP) algorithm [9]. However, these methods require prior knowledge for optimal operation. On the other hand, convex optimization-based algorithms, including ISTA [10], fast iterative shrinkage-thresholding algorithm (FISTA) [11], and alternating direction method of multipliers (ADMM) [12], exhibit robust reconstruction capabilities. Nonetheless, tuning parameters such as regularization weights and penalty factors within the ADMM algorithm remains challenging and often relies on empirical approaches [13].

Deep learning-based approaches, leveraging the robust learning capabilities of neural networks, have demonstrated remarkable success in signal reconstruction [14,15]. Mousavi et al. proposed a compressed sensing (CS)-based image reconstruction algorithm that enhances noisy image reconstruction performance using stacked denoising auto-encoders (SDA) [16]. However, the SDA model relies on fully connected neural networks with extensive interlayer weights, resulting in high computational complexity. To mitigate this issue, a neural network architecture inspired by ISTA was developed for image reconstruction, offering both interpretability and competitive performance [17]. More recently, the ISTA deep reconstruction network (ISTADR-Net) was introduced for reconstructing wideband spectrum signals [18]. Additionally, robust ADMM (R-ADMM), which integrates deep learning into the ADMM framework, has demonstrated promising reconstruction results [13]. Despite these advancements, most existing methods have yet to fully incorporate the block sparsity features (BSFs) inherent in wideband spectrum signals during the reconstruction process [19].

After obtaining the reconstructed wideband signal through the reconstruction algorithm, WSS can be performed. Traditional WSS techniques typically transform time-domain wireless signals into the frequency domain to extract statistical metrics from frequency subbands for decision-making. Although these methods are conceptually straightforward, they often fail to deliver optimal performance in complex channel environments. In contrast, deep learning-based approaches, which exploit the inherent structural information of wireless signals [20], achieve superior sensing performance.

D. Uvaydov et al. proposed DeepSense, a real-time WSS framework that integrates deep learning with real-time closed-loop processing to directly handle I/Q data [21]. This framework incorporates spectrum sensing capabilities into the baseband processing of software-defined radios (SDR). To reduce the runtime of DeepSense, R. Mei et al. introduced the ParallelCNN architecture, which is particularly suited for resource-constrained environments [22]. Z. Chen et al. developed a method that employs the short-time Fourier transform (STFT) to convert received signals into two-dimensional time–frequency matrices, which are subsequently normalized into grayscale images and used as inputs for deep neural networks [23]. This approach enhances the representation of signal features. Additionally, a spectrum sensing method based on an enhanced deep learning classification approach was proposed, utilizing a hybrid architecture of convolutional neural networks (CNNs) and recurrent neural networks (RNNs) [24]. This method enhances the accuracy and efficiency of spectrum sensing by facilitating feature extraction and time-series information capture, excelling in dynamic spectrum sensing. Furthermore, W. Zhang et al. introduced a WSS architecture based on the attention mechanism, referred to as “Spectrum Transformer” [25]. This model leverages multi-head self-attention mechanisms to capture both inter-band and intra-band correlations, thus achieving more accurate spectrum detection. Finally, R. Mei et al. introduced CNNWSS-Net, a novel network that utilizes the frequency-domain representation of wireless signals as input, improving detection accuracy without increasing computational complexity [18].

This paper primarily investigates the CSS technology in cognitive radio and decomposes it into a compressed reconstruction problem and a WSS problem. The key contributions are as follows: (1) Considering the BSF of wideband spectrum signals, we propose a novel neural network architecture called block sparsity feature enhancement ISTA networks (BEISTA-Net). This architecture replaces the proximal mapping in ISTA with multiple convolutional operations and incorporates a BSF extractor composed of one-dimensional convolutional kernels of varying sizes to extract the BSF of wideband spectrum signals. Additionally, the method employs coordinate attention (CA) [26] to enhance the BSF and suppress noise. (2) We propose a network named block sparse wideband spectrum sensing network (BSWSS-Net) for WSS. BSWSS-Net utilizes one-dimensional convolutional kernels of multiple sizes to extract low-level structural information from wideband spectrum signals and fuses these features through fully connected layers to effectively capture channel state information. (3) Extensive experiments are conducted on simulated spectrum data across different sparsity levels and SNRs. Numerical results demonstrate that the BEISTA-Net outperforms existing reconstruction algorithms in terms of reconstruction accuracy. Furthermore, compared to current deep learning-based WSS algorithms, the proposed BSWSS-Net reduces the number of floating point operations by at least 50% while maintaining detection accuracy.

The remainder of this paper is organized as follows: Section 2 introduces the system model. Section 3 presents the architecture and training methodology of BEISTA-Net and BSWSS-Net. Section 4 describes the datasets and performance metrics. Experiment results are provided in Section 5. Finally, the work of this paper is summarized in Section 6.

## 2. System Model

**Figure 1:**
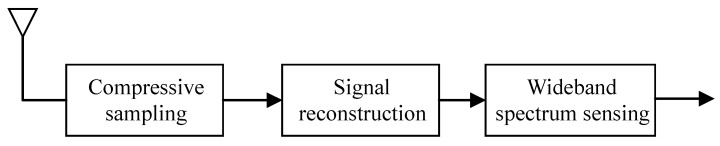
Block diagram of CSS.

As illustrated in Figure 1, the CSS problem can be decomposed into three key stages: compressive sampling, signal reconstruction, and WSS. Each of these stages is elaborated on in detail in this section.

### 2.1. Compressive Sampling

**Figure 2:**
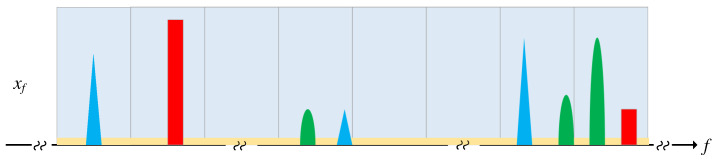
Block sparsity of wideband spectrum signals.

As illustrated in Figure 2, each primary user (PU) typically occupies a continuous segment of the spectrum in practice [27,28]. Furthermore, the sampled spectrum consists of blocks that are either occupied by continuous non-zero elements or occupied by continuous zero elements, thereby forming a block sparsity structure.

Wideband spectrum signals $x_{f}$ can be divided into *J* blocks in the frequency domain because of BSF and can be expressed as

(1) $$x_{f}={\left[\underset{x_{1}^{⊤}}{\underset{︸}{x_{1,1}\cdots x_{I,1}}}\underset{x_{2}^{⊤}}{\underset{︸}{x_{1,2}\cdots x_{I,2}}}\cdots \underset{x_{J}^{⊤}}{\underset{︸}{x_{1,J}\cdots x_{I,J}}}\right]}^{⊤},$$

where each block consists of *I* elements, and $x_{i,j}\in C$.

This work employs a multi-coset sampler (MCS) for sampling wideband spectrum signals. The MCS consists of *P* parallel cosets, each uniformly sampling at a rate of $\frac{f_{s}}{L}=\frac{1}{LT}$, with a sampling time offset of $c_{p}T$, and L≫P. Here, $c_{p}$ is chosen from the integer set $\left\{0,1,\ldots ,L-1\right\}$, and $f_{s}=\frac{1}{T}$ is the nyquist sampling rate. The compression ratio β is defined as P/L.

The compressive sampling of MCS can be expressed as

(2) Y=ΦX+E,

where $Y\in C^{P\times M}$, $X\in C^{L\times M}$ is the sparse representation of the original signal in the frequency domain and $E\in C^{P\times M}$ is the noise matrix. $\Phi \in C^{P\times L}$ is the observation matrix, with its elements denoted as $\Phi_{p,l}=\frac{1}{L\cdot T_{s}}\exp\left(\frac{j2\pi \cdot c_{p}\cdot l}{L}\right)$. Please note that X is obtained by reshaping $x_{f}$.

### 2.2. Compressed Signal Reconstruction

The compressed signal reconstruction is introduced and formulated as an iterative problem based on ISTA. The reconstruction of the compressed signal can be formulated as a convex optimization problem, which is expressed as

(3) $$\min_{X}\frac{1}{2}{\left\|\Phi X-Y\right\|}_{2}^{2}+\lambda \|X{\|}_{1},$$

where λ is the preset regularization parameter.

The ISTA is a popular first-order proximal algorithm that is well-suited for solving large-scale linear inverse problems. Specifically, the ISTA iteratively solves the above convex optimization problem through the following steps

(4) $$R^{\left(k\right)}=X^{\left(k-1\right)}-\rho \Phi^{⊤}\left(\Phi X^{\left(k-1\right)}-Y\right),$$

(5) $$X^{\left(k\right)}=\min_{X}\frac{1}{2}\|X-R^{\left(k\right)}{\|}_{2}^{2}\;+\;\lambda \|X{\|}_{1},$$

where *k* is the number of iterations of ISTA, and ρ is the step size of the algorithm.

Equation (5) is a special case of the so-called proximal mapping [17]. However, the ISTA requires careful parameter selection.

### 2.3. Wideband Spectrum Sensing

Once the reconstructed signal $\hat{X}$ is obtained by solving the above problem, WSS methods can be employed to determine the channel occupancy status. Both traditional spectrum energy detectors and deep learning-based methods can effectively address this problem. The spectrum energy detector determines the occupancy status of a subband by comparing its frequency-domain energy with a threshold τ. The energy of the $n_{c}$-th subband can be expressed as

(6) $$E_{n_{c}}=\frac{1}{N_{sc}}\sum_{n=1}^{N_{sc}}{\left|{\hat{x}}_{f}\left(\left(n_{c}-1\right)*N_{sc}+n\right)\right|}^{2},n_{c}=1,2,\cdots ,N_{c},$$

where $N_{c}$ denotes the number of the sub-channel, $N_{sc}\;=\;\frac{N}{N_{c}}$ denotes the number of samples per sub-channel. If $E_{n_{c}}>\tau$, the $n_{c}$-th channel is determined as occupied by a PU.

Deep learning-based methods transform the WSS problem into a multi-label classification problem. The reconstructed signal is used as input to a neural network to determine the occupancy status of each sub-channel. We note that deep learning-based methods can leverage more information from the reconstructed signal, thus achieving higher sensing accuracy.

## 3. The Proposed CSS Method

The design of the proposed CSS method is presented in this section, which consists of two key components: BEISTA-Net and BSWSS-Net. BEISTA-Net is designed for reconstructing compressed signals, while BSWSS-Net is designed for WSS.

### 3.1. Beista-Net Architecture

BEISTA-Net is introduced, which is a novel deep learning framework designed for wideband spectrum signal reconstruction. The framework incorporates BSF to enhance reconstruction performance. The key idea behind this approach is to map the iterative steps of ISTA onto a deep network framework, where each stage corresponds to one iteration of the traditional ISTA algorithm. At each stage, the network employs one-dimensional convolutional kernels of varying sizes to extract the BSF from wideband spectrum signals. Additionally, the CA mechanism is integrated at each stage to further enhance the extraction of BSF.

**Figure 3:**
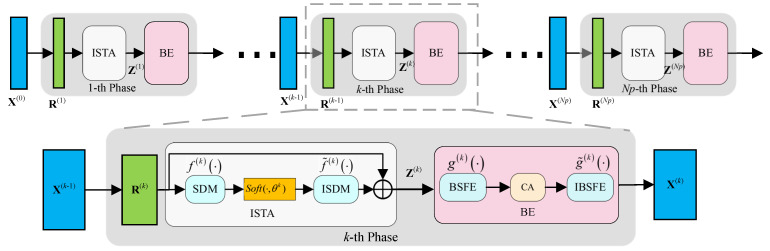
The proposed BEISTA-Net is composed of three components: the gradient update module $R^{\left(k\right)}$, the non-linear transformation module $Z^{\left(k\right)}$, and the feature enhancement module $X^{\left(k\right)}$. SDM denotes forward sparse domain mapping, while ISDM denotes inverse sparse domain mapping. BSFE denotes block sparse feature extraction, and IBSFE denotes inverse block sparse feature extraction.

As shown in Figure 3, BEISTA-Net consists of three components, with arrows indicating the direction of data flow. The components include the $R^{\left(k\right)}$ module, $Z^{\left(k\right)}$ module, and $X^{\left(k\right)}$ module. The $R^{\left(k\right)}$ module generates the immediate reconstruction result [17]. The $Z^{\left(k\right)}$ module performs a soft-thresholding process that removes less important parts from $R^{\left(k\right)}$. The $X^{\left(k\right)}$ module extracts and enhances the BSF from $Z^{\left(k\right)}$ to obtain accurate reconstruction results.

$R^{\left(k\right)}$**Module:** This module corresponds to Equation (4) and is used to generate the instantaneous reconstruction result $R^{\left(k\right)}$. Note that $\Phi^{⊤}\left(\Phi X^{\left(k-1\right)}-Y\right)$ is actually the gradient of the data-fidelity term $\frac{1}{2}{\left\|\Phi X-Y\right\|}_{2}^{2}$. To improve the reconstruction performance and increase its capacity, we permit the step size ρ to vary during iterations. The input to this layer is $X^{\left(k-1\right)}$, and the output is defined as

(7) $$R^{\left(k\right)}=X^{\left(k-1\right)}-\rho^{\left(k\right)}\Phi^{⊤}\left(\Phi X^{\left(k-1\right)}-Y\right).$$

$Z^{\left(k\right)}$**Module:** This module addresses the problem in Equation (5) using a neural network approach. BEISTA-Net employs a non-linear transformation function $f\left(\cdot \right)$ to transform $R^{\left(k\right)}$ into the sparse domain, rather than directly performing subsequent operations in a fixed sparse domain. The parameters of this transformation function are learnable. We use a combination of two symmetric linear convolution operators, with ReLU units added in between, to implement proximal mapping [29,30]. Additionally, a residual component is introduced to facilitate direct information transmission. The output of this module can be represented as

(8) $$Z^{\left(k\right)}=\alpha^{\left(k\right)}{\tilde{f}}^{\left(k\right)}\left(soft\left(f^{\left(k\right)}\left(R^{\left(k\right)}\right),\theta^{\left(k\right)}\right)\right)+\left(1-\alpha^{\left(k\right)}\right)R^{\left(k\right)},$$

where $\alpha^{\left(k\right)}$ represents the ratio of the residual component to the computational component, with a range of 0 to 1, and $soft\left(z,\theta^{\left(k\right)}\right)$ is the soft-thresholding function, which can be expressed as $soft\left(z,\theta^{\left(k\right)}\right)=\mathrm{sign}(z)\mathrm{ReLU}\left(\left|z\right|-\theta^{\left(k\right)}\right)$.

**Figure 4:**
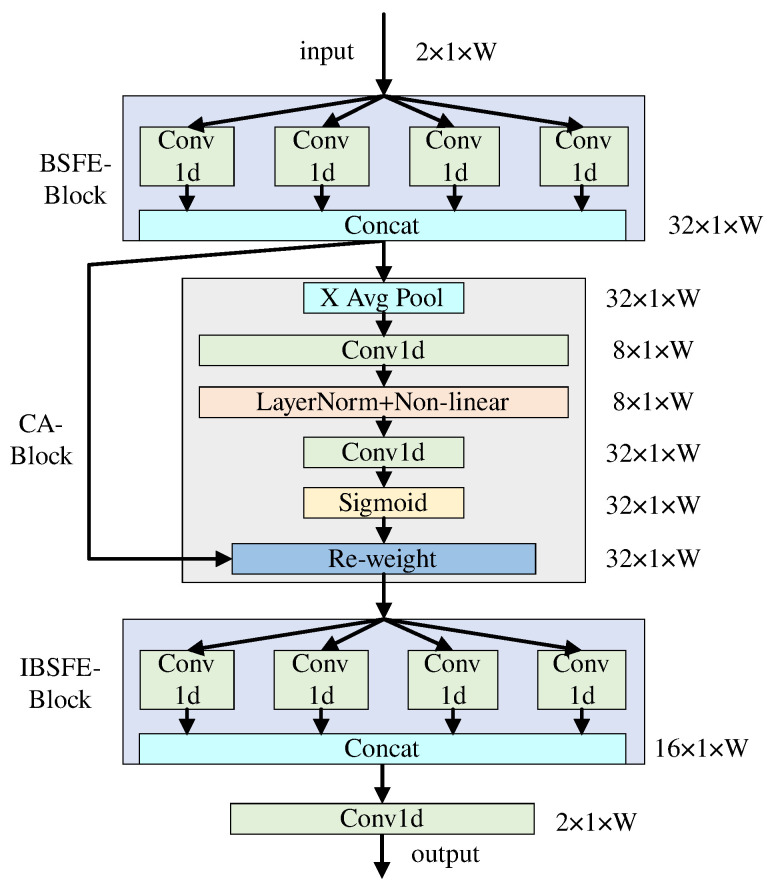
The BE-block consists of the BSFE-Block, CA-Block, and IBSFE-Block. The BSFE-Block denotes block sparsity feature extraction, IBSFE-Block denotes inverse block sparsity feature extraction. The BSFE-Block consists of 4 sets of convolutional kernels (each with sizes of 1 × 7, 1 × 15, 1 × 23, 1 × 31), where each set of kernels expands the 2-channel data to 8-channel data. X Avg Pool represents average pooling along the horizontal dimension. Conv1d denotes reducing the number of channels to decrease computational complexity. LayerNorm+Non-linear indicates layer normalization and non-linear operations. Re-weight represents matrix multiplication with the obtained sparse matrix. The IBSFE-Block is symmetrically structured with the BSFE-Block.

$X^{\left(k\right)}$**Module:** The network expansion diagram of the $X^{\left(k\right)}$ module is shown in Figure 4. First, this module extracts the BSF of the wideband spectrum signals by the BSFE-Block. Then, it enhances these features by the CA-Block. Finally, it maps the enhanced signals back to the original dimensions by the IBSFE-Block. The CA-Block transforms the captured features into corresponding coefficients and multiplies them with their original data to obtain the enhanced signals. The output of the feature enhancement module is

(9) $$X^{\left(k\right)}={\tilde{g}}^{\left(k\right)}\left(A⊙g^{\left(k\right)}\left(Z^{\left(k\right)}\right)\right),$$

where A is the coefficient matrix generated by CA, $g^{\left(k\right)}\left(\cdot \right)$ is the function for extracting BSF, ${\tilde{g}}^{\left(k\right)}\left(\cdot \right)$ is the reverse process of $g^{\left(k\right)}\left(\cdot \right)$.

The BSF of wideband spectrum signals is analogous to texture features in images, which enables the extraction of the BSF using convolutional kernels. The BSF exhibits a one-dimensional distribution, which allows for the efficient use of one-dimensional convolutional kernels for its extraction. Moreover, in practical applications, blocks may vary in scale, and employing multiple one-dimensional convolutional kernels of different sizes can more accurately extract features. The size of the convolutional kernels can be flexibly adjusted to extract BSF at different scales. Additionally, the CA in this work is a lightweight attention mechanism that only involves one-dimensional convolution, resulting in significantly reduced computational complexity compared to self-attention mechanisms. Note that when using the $X^{\left(k\right)}$ module, we first convert the two-dimensional wideband spectrum signal into a one-dimensional form. This allows for better extraction of the one-dimensional BSF.

### 3.2. BSWSS-Net Architecture

**Figure 5:**
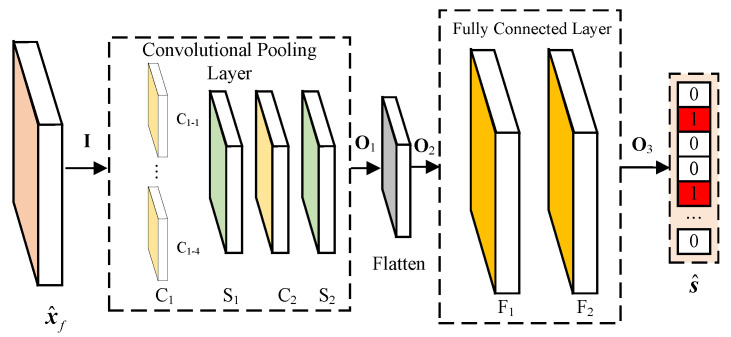
BSWSS-Net.

The multi-label classification problem allows each input sample to belong to multiple categories. In WSS, this implies the presence of multiple vacant subbands within the entire frequency band. To address this, we designed a multi-label classifier, BSWSS-Net, which outputs an $N_{c}$-dimensional vector indicating the occupancy status of $N_{c}$ channels. The structure and hyperparameters of BSWSS-Net are illustrated in Figure 5 and detailed in Table 1, respectively. The newly proposed BSWSS-Net extracts local features of wideband spectrum signals using four one-dimensional convolution kernels of different sizes. The global features are then fused through fully connected layers, ultimately yielding the sub-channel state. The use of convolution kernels of different sizes makes it easier to extract the sparse block characteristics of wideband spectrum signals. Additionally, by balancing the number of parameters in the convolutional and fully connected layers, the overall parameter count of the network is effectively reduced.

The input data I is sequentially processed through convolutional pooling layers and fully connected layers, ultimately yielding the channel occupancy status output. The output of the convolutional pooling layer can be represented as

(10) $$O_{1}=f_{\mathrm{avg}}\left(f_{L}\left(W_{C_{2}}*f_{\mathrm{avg}}\left(f_{L}\left(W_{C_{1}}*I+b_{C_{1}}\right)\right)+b_{C_{2}}\right)\right),$$

where $W_{C_{i}}$ is the convolutional filters of $C_{i}$ layer, $b_{C_{i}}$ is the bias of $C_{i}$ layer, $f_{\mathrm{avg}}$ donates the average pooling function, and $f_{L}$ donates the LeakyReLU activation function,

(11) $$\left.f_{L}\left(x\right)=\left\{\begin{array}{cc} x, & \mathrm{if}\;x\geq 0\\ 0.1\times x, & \mathrm{otherwise} \end{array}\right.\right..$$

Then, $O_{1}$ is flattened into a vector, and the flattening process can be represented as

(12) $$O_{2}=\mathrm{Flatten}\left(O_{1}\right).$$

The local feature vector is processed through fully connected layers to obtain global features, and the output is generated through an activation function. The output of the fully connected layer can be represented as

(13) $$\hat{s}=f_{\mathrm{Sigmoid}}\left(W_{F_{2}}*\left(W_{F_{1}}*O_{2}+b_{F_{1}}\right)+b_{F_{2}}\right),$$

where $W_{F_{i}}$ is the weights of the fully connected layer $F_{i}$ layer, $b_{F_{i}}$ is the bias of the fully connected $F_{i}$ layer, and the activation function can be represents as

(14) $$f_{\mathrm{Sigmoid}}=\frac{1}{1+e^{-x}}.$$

### 3.3. Training Methodology

The training of the CSS method is performed in two stages. The first stage involves training the BEISTA-Net. Considering both computational complexity and performance, the number of network iterations is set to four. Given training data ${\left\{\left(Y_{i},X_{i}\right)\right\}}_{i=1}^{N_{CS}}$, BEISTA-Net takes compressed measurements $Y_{i}$ as input and generates the reconstruction result $X_{i}^{\left(N_{p}\right)}$ as output. Similar to traditional ISTA, BEISTA-Net requires an initial value, which is set as $X^{\left(0\right)}=\Phi^{⊤}Y$. During training, we aim to minimize the difference between $X_{i}^{\left(N_{p}\right)}$ and $X_{i}$ while satisfying the symmetry constraint ${\tilde{f}}^{\left(k\right)}\circ f^{\left(k\right)}=I;\forall k=1,\ldots ,N_{p}$ [17]. Therefore, we incorporate a regularization term to enforce the symmetry constraint, in addition to the relative mean squared error (r-MSE) term, which can help prevent overfitting. The final loss function is expressed as

(15) $$L_{CS}=L_{discrepancy}+\gamma L_{constraint},$$

with

(16) $$\left\{\begin{array}{c} L_{discrepancy}=\frac{1}{N_{CS}}\sum_{i=1}^{N_{CS}}\frac{\|X_{i}^{\left(N_{p}\right)}-X_{i}{\|}_{2}^{2}}{\|X_{i}{\|}_{2}^{2}}\\ L_{constraint}=\frac{1}{N_{CS}N_{p}}\sum_{i=1}^{N_{CS}}\sum_{k=1}^{N_{p}}\frac{\|{\tilde{f}}^{\left(k\right)}\left(f^{\left(k\right)}\left(X_{i}\right)\right)-X_{i}{\|}_{2}^{2}}{\|X_{i}{\|}_{2}^{2}}, \end{array}\right.$$

where $N_{CS}$, $N_{p}$, γ are the size of the training dataset, the number of network layers, and the regularization parameter, respectively. In the experiments, γ is set to 0.001.

Typically, the collected wideband spectrum signals are in a complex form. The frequency domain representation of the wideband spectrum signals is directly utilized as the training data. To better adapt to the neural network, we use the real and imaginary parts of the frequency domain representation as two separate channels for input during training. This approach yields satisfactory performance as the powerful learning capability of neural networks.

When training BEISTA-Net, we use the Adam optimizer with a maximum of 100 epochs and stop the training process when an increase in the loss value on the test set is detected. The learning rate is initially set to 0.001 for the first 30 epochs and then reduced to 0.0001.

Next, the BSWSS-Net is trained using the reconstructed signals to predict $\hat{s}$. The training data are represented as,

(17) $$\begin{array}{c} \left(I,S\right)=\left\{\left(i^{\left(1\right)},s^{\left(1\right)}\right),\cdots ,\left(i^{\left(n_{WSS}\right)},s^{\left(n_{WSS}\right)}\right),\cdots ,\left(i^{\left(N_{WSS}\right)},s^{\left(N_{WSS}\right)}\right)\right\}, \end{array}$$

where $i^{\left(N_{WSS}\right)}$ represents the input data for the neural network, $N_{WSS}$ represents the size of the training dataset, $s^{\left(n_{WSS}\right)}\;=\;\left[s_{1}^{\left(n_{WSS}\right)},\cdots ,s_{n_{c}}^{\left(n_{WSS}\right)},\cdots ,s_{N_{c}}^{\left(n_{WSS}\right)}\right]$ represents $N_{c}$ labels, $s_{n_{c}}^{\left(n_{WSS}\right)}\;\in \;\left\{0,1\right\}$. The binary cross-entropy loss function is used as the loss function for BSWSS-Net and can be expressed as

(18) $$L_{WSS}=-\frac{1}{N_{c}N_{WSS}}\sum_{n_{WSS}=1}^{N_{WSS}}\sum_{n_{c}=1}^{Nc}\left(s_{n_{c}}^{\left(n_{WSS}\right)}\log\left({\hat{s}}_{n_{c}}^{\left(n_{WSS}\right)}\right)+\left(1-s_{n_{c}}^{\left(n_{WSS}\right)}\right)\left(\log\left(1-{\hat{s}}_{n_{c}}^{\left(n_{WSS}\right)}\right)\right)\right).$$

When training BSWSS-Net, we use the Adam optimizer with a maximum of 200 epochs and stop the training process when an increase in the loss value on the test set is detected. The learning rate is initially set to 0.001 for the first 30 epochs and then reduced to 0.0001. To prevent overfitting, we add dropout layers between the fully connected layers, with a dropout rate set to 0.05.

## 4. Datasets and Performance Metrics

The specific parameters of the two datasets and the evaluation metrics used to assess network performance are introduced.

### 4.1. Two Datasets

This study employs two datasets, TVWS Dataset and NR Dataset, generated in MATLAB R2022b to train and evaluate the proposed method. Each dataset contains 200,000 samples, each sample contains 2048 I/Q sampling points, and the SNR of the signals is uniformly distributed between [−10, 10] dB.

The TVWS signal occupies a total bandwidth of 320 MHz, consisting of 160 non-overlapping sub-channels, each with a bandwidth of 2 MHz. The signal model is expressed as

(19) $$x\left(t\right)=\sum_{k=1}^{K}\sqrt{E_{k}B}\;\sin c\left[B\left(t-t_{k}\right)\right]e^{j2\pi f_{k}t}+n\left(t\right),$$

where x(t)∈F=[0,320] MHz is divided into L=32 channels, *K* denotes the number of users, $E_{k}\in \left[0.3,1\right]$ represents the energy of each user (based on parameters in [13]), $t_{k}$ is the time offset of each user, $f_{k}$ indicates the carrying frequency of each user, n(t) represents additive white Gaussian noise, and *B* is the bandwidth of each user, set to 2 MHz. The sparsity level of the TVWS dataset is defined as

(20) $$\mu_{TVWS}=\frac{2K}{320}.$$

The NR signal occupies a total bandwidth of 400 MHz, consisting of 132 non-overlapping sub-channels, each with a bandwidth of 2.88 MHz. This dataset is created by simulating 5G NR link transmissions in the physical uplink shared channel (PUSCH). Within this dataset, the subcarrier spacing of the signals is 120 kHz and each resource block occupies a 1.44 MHz channel. Each user occupies two resource blocks. The sparsity level μ of the NR dataset is defined as

(21) $$\mu_{NR}=\frac{2.88K}{400}.$$

### 4.2. Performance Metrics

The evaluation metrics for the proposed networks are introduced. For BEISTA-Net, the r-MSE is used as the evaluation metric. A lower r-MSE indicates that the reconstructed signal more closely resembles the original signal. For BSWSS-Net, detection probability ($P_{d}$) and false alarm probability ($P_{f}$) are employed as the evaluation metric. Generally, a higher $P_{d}$ and a lower $P_{f}$ are desirable.

The r-MSE is defined as

(22) $$\mathrm{r-MSE}=\frac{{∥X_{r}-X∥}_{2}^{2}}{{∥X∥}_{2}^{2}},$$

where X is the original signal and $X_{r}$ is the reconstructed signal.

The detection probability is defined as the ratio of correctly detected samples (true positives) to the total number of actual positive samples, which can be expressed as

(23) $$P_{d}=\frac{\mathrm{True}\;\mathrm{Positives}}{\mathrm{True}\;\mathrm{Positives}\;+\mathrm{False}\;\mathrm{Negatives}},$$

where the True Positives is the number of correctly detected positive samples, and False Negatives is the number of positive samples that were incorrectly classified as negative.

The false alarm probability is defined as the ratio of incorrectly detected negative samples (false positives) to the total number of actual negative samples, which can be expressed as

(24) $$P_{f}=\frac{\mathrm{False}\;\mathrm{Positives}}{\mathrm{True}\;\mathrm{Negatives}\;+\mathrm{False}\;\mathrm{Positives}},$$

where False Positives is the number of negative samples incorrectly classified as positive, and True Negatives is the number of correctly classified negative samples.

In cognitive radio, it is generally required that $P_{d}>0.9$ and $P_{f}<0.1$.

## 5. Experiment Results

The performance of the proposed method is evaluated on the NR dataset and TVWS dataset. The evaluation includes the reconstruction performance of BEISTA-Net, the sensing performance of BSWSS-Net, and the sensing performance of the joint CSS method.

### 5.1. BEISTA-Net

#### 5.1.1. Reconstruction Accuracy

**Figure 6:**
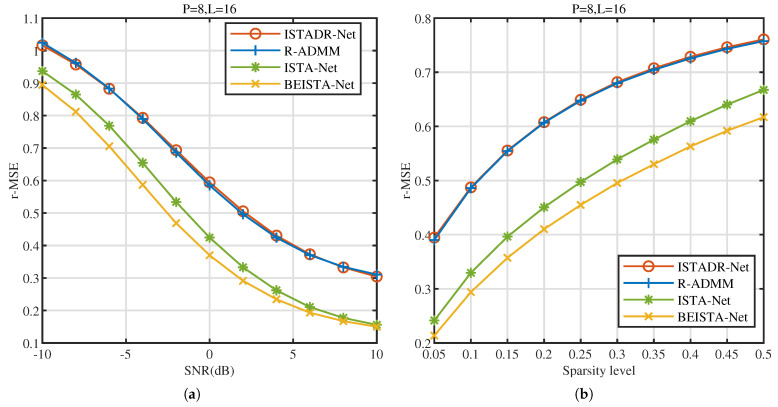
TVWS dataset. (**a**) r-MSE versus SNR (dB) of ISTADR-Net, R-ADMM, ISTA-Net, and BEISTA-Net when P=8, L=16, and $\mu_{TVWS}\in \left(0,0.5\right]$. (**b**) r-MSE versus sparsity level of ISTADR-Net, R-ADMM, ISTA-Net, and BEISTA-Net when P=8, L=16, and SNR ∈[−10,10] dB.

As shown in Figure 6a, we compare the r-MSE of BEISTA-Net with that of ISTADR-Net [18], R-ADMM [13], and ISTA-Net under various SNR conditions in the TVWS dataset. From this figure, it is evident that as SNR increases, the r-MSE of all four algorithms decreases. When SNR=10dB, the r-MSE of BEISTA-Net is even lower than 0.2. BEISTA-Net and ISTA-Net significantly outperform the other two algorithms across all SNR conditions. Furthermore, BEISTA-Net demonstrates a substantial improvement in performance over ISTA-Net. These findings suggest that the BE-Block effectively extracts and enhances the BSF of wideband spectrum signals, improving reconstruction accuracy.

As shown in Figure 6b, we compare the r-MSE of BEISTA-Net, ISTADR-Net [18], R-ADMM [13], and ISTA-Net under different sparsity level in TVWS dataset. As the sparsity level increases, the r-MSE of all algorithms also increases. The results demonstrate that under different sparsity levels, both BEISTA-Net and ISTA-Net significantly outperform the other two algorithms. Furthermore, BEISTA-Net exhibits a substantial improvement in performance over ISTA-Net. As the sparsity level increases, the performance of BEISTA-Net gradually approaches that of the other three algorithms. This is because the performance enhancement of BEISTA-Net relies on the sparsity of the signal. Generally, the sparser the signal, the greater the performance improvement achieved by BEISTA-Net.

**Figure 7:**
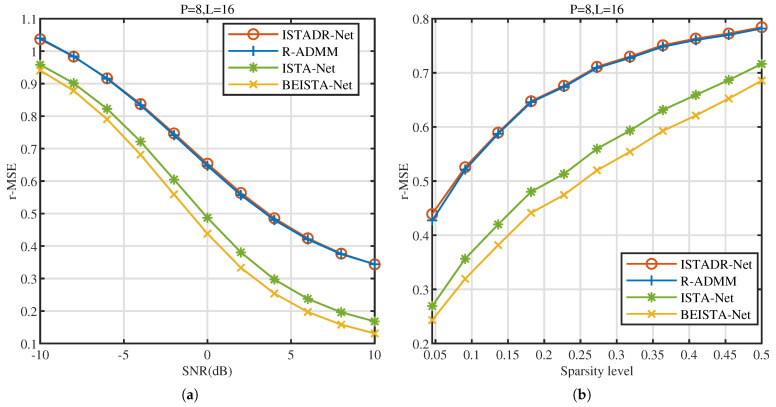
NR dataset. (**a**) r-MSE versus SNR (dB) of ISTADR-Net, R-ADMM, ISTA-Net, and BEISTA-Net when P=8, L=16, and $\mu_{NR}\in \left(0,0.5\right]$. (**b**) r-MSE versus sparsity level of ISTADR-Net, R-ADMM, ISTA-Net, and BEISTA-Net when P=8, L=16, and SNR∈[−10,10]dB.

As shown in Figure 7, we compare the performance of BEISTA-Net with that of the other three algorithms under different SNR conditions and sparsity levels in the NR dataset. From these figures, it can be observed that the proposed BEISTA-Net outperforms the comparison algorithms across various SNRs and sparsity levels in the NR dataset. This indicates that the proposed BEISTA-Net demonstrates strong robustness.

#### 5.1.2. Complexity Analysis of Reconstruction Algorithms

To compare the computational complexity of different reconstruction algorithms, the number of floating point operations (FLOPs), running time, video memory usage, and the number of learnable parameters are evaluated for BEISTA-Net, ISTADR-Net [18], R-ADMM [13], and ISTA-Net. It is important to note that the computational complexity of the reconstruction algorithm only depends on the size of input and output. Since the size is identical in the TVWS dataset and NR dataset during reconstruction, the computational complexity of the reconstruction algorithm is the same in both datasets. Here, we use the parameters P=8, L=16, and M=128. We implemented these algorithms on a PC equipped with an Intel Core i9-13900KF 3 GHz CPU and two Nvidia GeForce RTX 4080 Super GPUs to evaluate their runtime. As shown in Table 2, compared to ISTADR-Net and R-ADMM, the FLOPs and the number of learnable parameters required by ISTA-Net and BEISTA-Net are significantly larger. This is because ISTA-Net and BEISTA-Net rely on a large number of convolutional kernels to extract features, while ISTADR-Net and R-ADMM rely on multiple iterations to reconstruct signals. However, the runtime required by ISTA-Net and BEISTA-Net is shorter because the neural network has a higher degree of parallelism in execution, significantly reducing the computation time. Compared to ISTA-Net, the runtime required by BEISTA-Net is approximately 1.9 times greater due to the inclusion of BE-Block. However, the FLOPs required by BEISTA-Net only increased slightly. The video memory usage of BEISTA-Net is the highest among the four algorithms, which is attributed to its large number of learnable parameters. BEISTA-Net effectively improves the reconstruction accuracy of the WSS algorithm at the cost of an acceptable increase in computational complexity. However, due to the higher video memory usage of BEISTA-Net, its deployment on embedded devices incurs additional resource consumption.

### 5.2. BSWSS-Net

#### 5.2.1. Sense Accuracy

**Figure 8:**
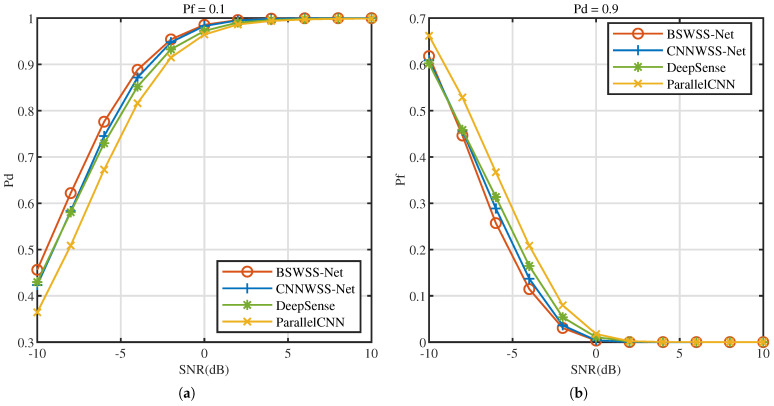
TVWS dataset with $\mu_{TVWS}\in \left(0,0.5\right]$. (**a**) $P_{d}$ versus SNR (dB) of BSWSS-Net, CNNWSS, DeepSense, and ParallelCNN when $P_{f}=0.1$. (**b**) $P_{f}$ versus SNR (dB) of BSWSS-Net, CNNWSS-Net, DeepSense, and ParallelCNN when $P_{d}=0.9$.

As shown in Figure 8a, we compare $P_{d}$ of BSWSS-Net, CNNWSS-Net [18], DeepSense [21], and ParallelCNN [22] under various SNR conditions at $P_{f}=0.1$ in the TVWS dataset. As shown in Figure 8b, we compare $P_{f}$ of BSWSS-Net, CNNWSS-Net, DeepSense, and ParallelCNN under various SNR conditions at $P_{d}=0.9$ in the TVWS dataset. As SNR increases, $P_{d}$ increases, and the $P_{f}$ decreases, indicating an improvement in algorithm performance. From these figures, it can be observed that the performance of BSWSS-Net and CNNWSS-Net is similar and much better than DeepSense and ParallelCNN. When SNR>−3dB, BSWSS-Net can meet the requirement of $P_{d}>0.9$, while $P_{f}<0.1$. These results indicate that, under varying levels of noise, BSWSS-Net can more effectively identify spectrum holes with a higher probability, thereby improving the efficiency and reliability of spectrum sensing.

**Figure 9:**
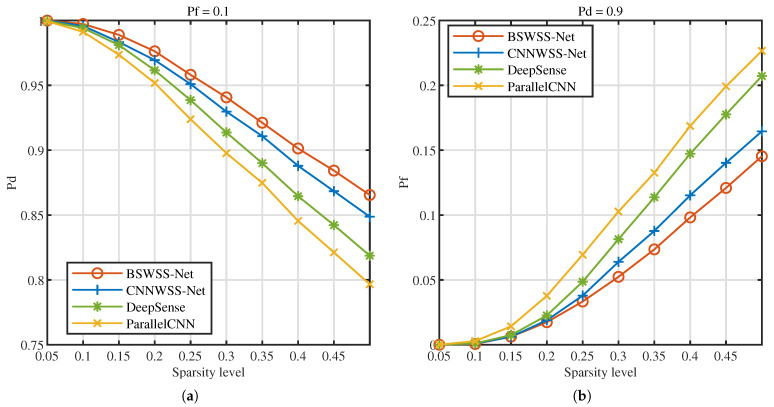
TVWS dataset with SNR ∈[−10,10] dB. (**a**) $P_{d}$ versus sparsity level of BSWSS-Net, CNNWSS, DeepSense, and ParallelCNN when $P_{f}=0.1$. (**b**) $P_{f}$ versus sparsity level of BSWSS-Net, CNNWSS-Net, DeepSense, and ParallelCNN when $P_{d}=0.9$.

Next, the impact of signal sparsity level on detection performance is evaluated in the TVWS dataset. Figure 9a illustrates the $P_{d}$ versus sparsity level at $P_{f}=0.1$, Figure 9b illustrates the $P_{f}$ versus sparsity level at $P_{d}=0.9$. It is distinctly observable that with the augmentation of signal sparsity, the detection probabilities of the four spectrum-sensing algorithms diminish, while the false alarm probabilities escalate. This phenomenon can be attributed to the fact that higher signal sparsity implies a greater number of users within the same bandwidth, leading to increased signal complexity, which consequently degrades detection performance. At a sparsity level of 0.05, the detection probabilities of the several algorithms approximate 1, and the false alarm probabilities are nearly 0. When the sparsity reaches 0.5, the BSWSS-Net achieves a detection probability of approximately 0.86 and a false alarm probability of about 0.15. Under identical conditions, CNNWSS-Net, DeepSense, and ParallelCNN exhibit detection probabilities significantly lower than that of BSWSS-Net, and their false alarm probabilities are notably higher. In summary, the proposed BSWSS-Net attains excellent detection performance across varying sparsity levels.

**Figure 10:**
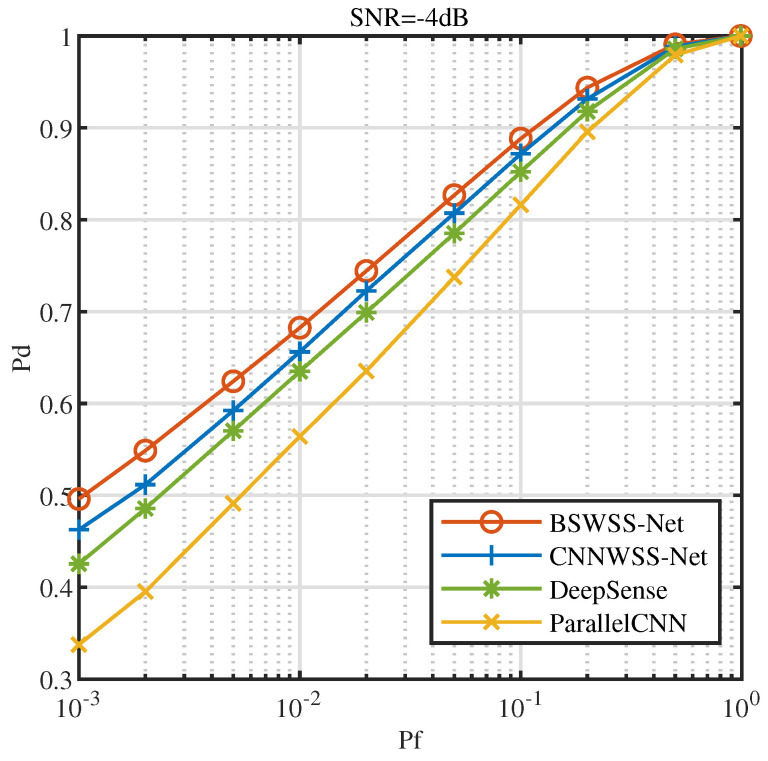
ROC curves when SNR=−4dB in TVWS dataset and $\mu_{TVWS}\in \left(0,0.5\right]$.

Figure 10 illustrates the receiver operating characteristics (ROC) at SNR=−4dB. It is evident that as the false alarm probability $P_{f}$ increases, the detection probability of all four algorithms also increases. Under the same conditions, BSWSS-Net consistently achieves the highest detection probability. Specifically, when $P_{f}=0.1$, the detection probability of BSWSS-Net nearly reaches 0.9, essentially meeting the requirements of cognitive radio. This observation clearly demonstrates that the detection performance of the BSWSS-Net algorithm surpasses that of the other three algorithms.

**Figure 11:**
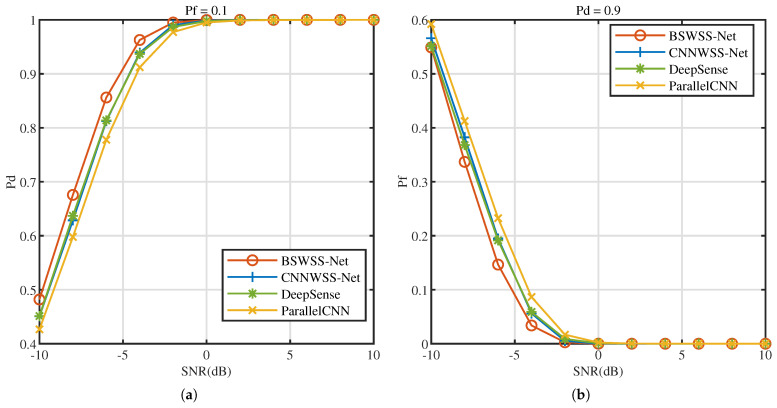
NR dataset with $\mu_{NR}\in \left(0,0.5\right]$. (**a**) $P_{d}$ versus SNR (dB) of BSWSS-Net, CNNWSS-Net, DeepSense, and ParallelCNN when $P_{f}=0.1$. (**b**) $P_{f}$ versus SNR (dB) of BSWSS-Net, CNNWSS-Net, DeepSense, and ParallelCNN when $P_{d}=0.9$.

**Figure 12:**
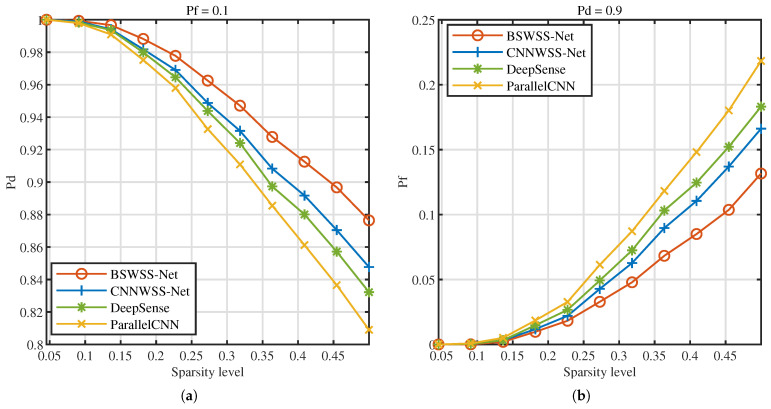
NR dataset with SNR ∈[−10,10] dB. (**a**) $P_{d}$ versus SNR (dB) of BSWSS-Net, CNNWSS, DeepSense, and ParallelCNN when $P_{f}=0.1$. (**b**) $P_{f}$ versus SNR (dB) of BSWSS-Net, CNNWSS-Net, DeepSense, and ParallelCNN when $P_{d}=0.9$.

**Figure 13:**
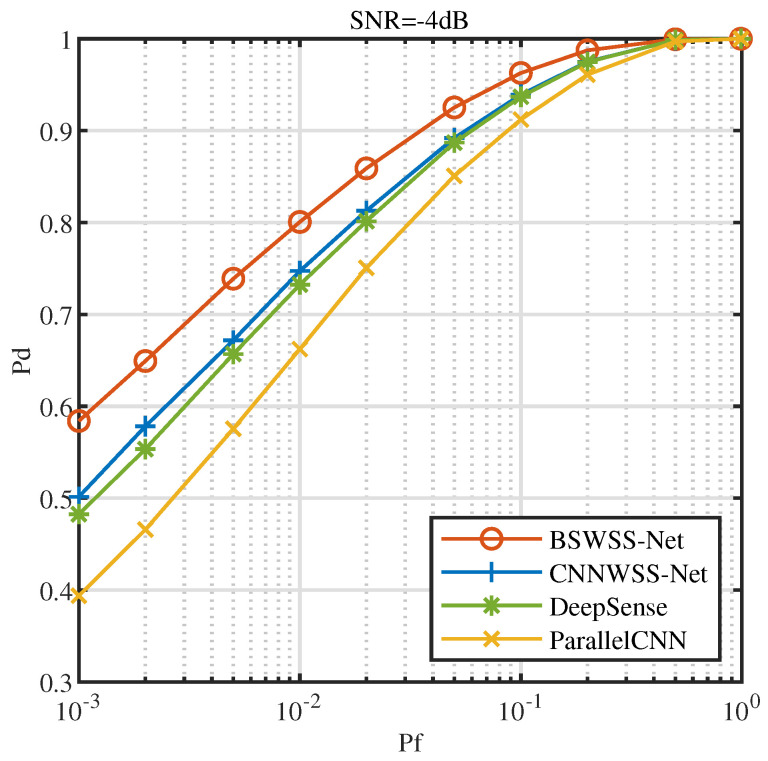
ROC curves when SNR=−4dB in NR dataset and $\mu_{NR}\in \left(0,0.5\right]$.

We also evaluate the performance of WSS algorithms in the NR dataset. Similarly, the impact of the SNR and sparsity level on detection performance, as well as ROC, were evaluated. From Figure 11, Figure 12 and Figure 13, it is evident that BSWSS-Net outperforms the other three algorithms. Specifically, under identical conditions, the proposed BSWSS-Net consistently achieves a higher detection probability and a lower false alarm probability compared to other methods. Compared to the results in the TVWS dataset, the four algorithms exhibit better performance in the NR dataset. This is because the size of the label in the NR dataset is smaller, which leads to better classification results. From the test results of the two datasets, it is evident that the proposed BSWSS-Net demonstrates excellent performance across various conditions, indicating the reliability of the algorithm.

#### 5.2.2. Complexity Analysis of WSS Algorithms

To compare the computational complexity of different WSS algorithms, the FLOPs, running time, video memory usage, and the number of learnable parameters are evaluated for DeepSense [21], ParallelCNN [22], CNNWSS-Net [18], and BSWSS-Net. In the TVWS dataset, the size of label $N_{c}=160$, while in the NR dataset, the size of label $N_{c}=132$. Thus, we evaluate the computational complexity in these two datasets separately. The input signal length is set to 2048 for both datasets. We implemented these algorithms on a PC equipped with an Intel Core i9-13900KF 3 GHz CPU and two Nvidia GeForce RTX 4080 Super GPUs to evaluate their runtime. As shown in Table 3, compared to the NR dataset, the number of FLOPs, the number of learnable parameters, video memory usage, and running time required by several algorithms are slightly higher when evaluated in the TVWS dataset. This is because the size of the label is slightly larger in the TVWS dataset. The FLOPs required by BSWSS-Net are approximately half of those required by the other three algorithms, with the fewest learnable parameters and video memory usage. Due to the low video memory usage of BSWSS-Net, it can be efficiently deployed on embedded devices.

### 5.3. Joint CSS Method

To compare the performance of different algorithms, the joint performance of the reconstruction and WSS algorithms is evaluated. BEISTA-Net+BSWSS-Net denotes that BEISTA-Net is employed for reconstructing signals, and BSWSS-Net is employed for WSS. ISTA-Net+DeepSense [21] denotes that ISTA-Net is employed for reconstructing signals, and DeepSense is employed for WSS. CSS-Net [18] denotes that ISTADR-Net is employed for reconstructing signals and CNNWSS-Net is employed for WSS. R-ADMM [13] +ParallelCNN [22] denotes that R-ADMM is employed for reconstructing signals and ParallelCNN is employed for WSS.

**Figure 14:**
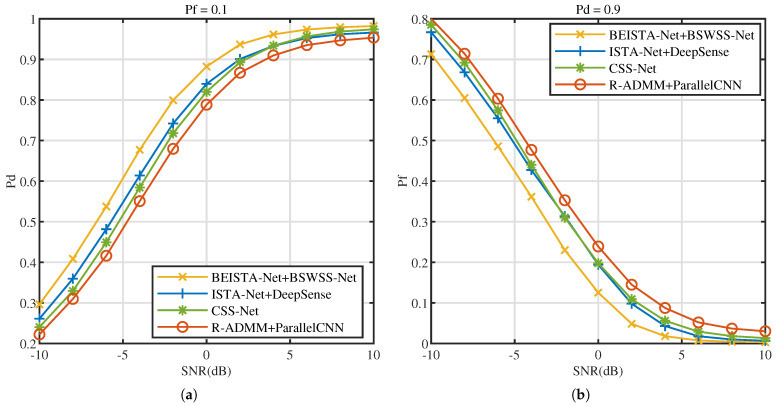
TVWS dataset with $\mu_{TVWS}\in \left(0,0.5\right]$, P=8, L=16. (**a**) $P_{d}$ versus SNR (dB) of BEISTA-Net+BSWSS-Net, ISTA-Net+DeepSense, CSS-Net, and R-ADMM+ParallelCNN when $P_{f}\;=\;0.1$. (**b**) $P_{f}$ versus SNR (dB) of BEISTA-Net+BSWSS-Net, ISTA-Net+DeepSense, CSS-Net, and R-ADMM+ParallelCNN when $P_{d}=0.9$.

We evaluate the performance in the TVWS dataset, with $\mu_{TVWS}\in \left(0,0.5\right]$ P=8 and L=16. The effect of SNR on the CSS algorithms is studied. Figure 14a illustrates the $P_{d}$ versus SNR at $P_{f}=0.1$, Figure 14b illustrates the $P_{f}$ versus SNR at $P_{d}=0.9$. As SNR increases, the performance of all four algorithms progressively improves. Notably, when SNR increases from −10 dB to 10 dB, the performance of BEISTA-Net+BSWSS-Net joint algorithm consistently surpasses that of the other three algorithms. When SNR>1dB, the detection probability can meet the requirement of $P_{d}>0.9$, while $P_{f}<0.1$. Compared to standalone WSS algorithms, the performance of CSS algorithms is slightly worse. This is because the reconstruction algorithm introduces some reconstruction errors, which affects the overall accuracy. The aforementioned experimental results demonstrate that the proposed BEISTA-Net+BSWSS-Net joint algorithm maintains robust detection performance under varying SNR conditions.

**Figure 15:**
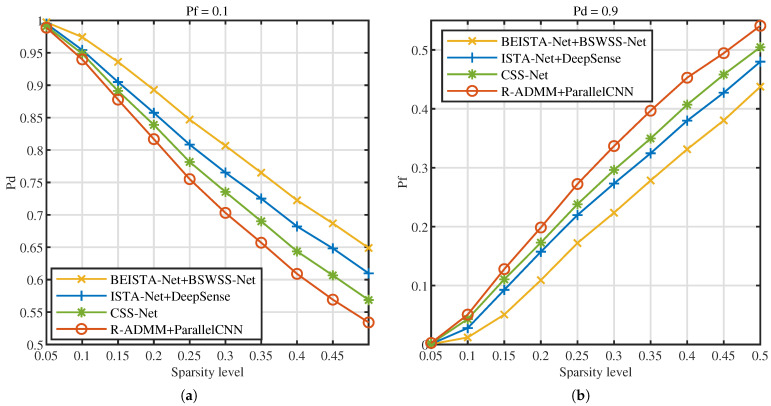
TVWS dataset with SNR ∈[−10,10] dB, P=8, L=16. (**a**) $P_{d}$ versus SNR (dB) of BEISTA-Net+BSWSS-Net, ISTA-Net+DeepSense, CSS-Net, and R-ADMM+ParallelCNN when $P_{f}=0.1$. (**b**) $P_{f}$ versus SNR (dB) of BEISTA-Net+BSWSS-Net, ISTA-Net+DeepSense, CSS-Net, and R-ADMM+ParallelCNN when $P_{d}=0.9$.

Next, the influence of signal sparsity levels on detection performance is investigated. Figure 15a illustrates the $P_{d}$ versus sparsity level at $P_{f}=0.1$, Figure 15b illustrates the $P_{f}$ versus sparsity level at $P_{d}=0.9$. As the sparsity level increases, the detection probabilities of the four joint algorithms decrease, while the false alarm probabilities rise. This phenomenon occurs because, with higher sparsity levels, the number of users contained in the signal increases, leading to greater signal complexity and, consequently, higher detection difficulty. Under the same sparsity level, the BEISTA-Net+BSWSS-Net joint algorithm achieves the highest detection probability and the lowest false alarm probability. Specifically, when the sparsity level is 0.05, the detection probability of the BEISTA-Net+BSWSS-Net joint algorithm is approximately 1, and the false alarm probability is nearly 0. When the sparsity level reaches 0.5, the detection probability of the BEISTA-Net+BSWSS-Net joint algorithm remains around 1, while the false alarm probability increases to approximately 0.45. Compared to the corresponding WSS algorithms, this represents a noticeable decline in detection performance, primarily due to the significantly reduced information obtained from compressive sampling, which degrades the quality of the reconstructed signal. Overall, the proposed joint CSS algorithm demonstrates robust performance across varying sparsity levels.

**Figure 16:**
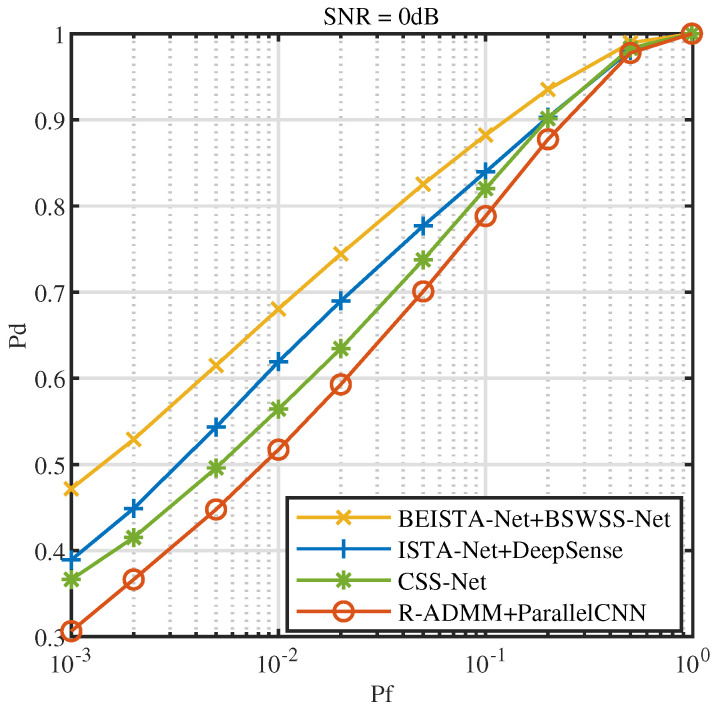
ROC curves when SNR=0dB in TVWS dataset, $\mu_{NR}\in \left(0,0.5\right]$, P=8, and L=16.

Figure 16 illustrates the ROC at SNR=0dB. From the figure, it is clear that BEISTA-Net+BSWSS-Net significantly outperforms ISTA-Net+DeepSense, CSS-Net, and R-ADMM+ParallelCNN. At $P_{f}=0.1$, $P_{d}$ for BEISTA-Net+BSWSS-Net is 88.23%, while ISTA-Net+DeepSense achieves 83.95%, CSS-Net achieves 82.02%, and R-ADMM+ParallelCNN achieves 78.82%. Compared to the current state-of-the-art algorithms, the $P_{d}$ is improved by 4 percentage points when $P_{f}=0.1$ and SNR=0dB.

**Figure 17:**
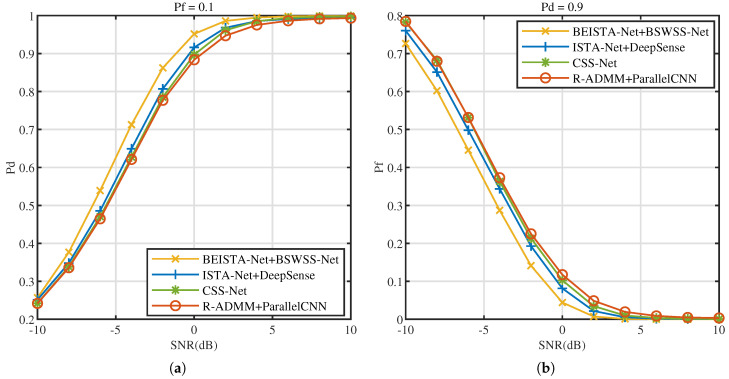
NR dataset with $\mu_{NR}\in \left(0,0.5\right]$, P=8, L=16. (**a**) $P_{d}$ versus SNR (dB) of BSWSS-Net, CNNWSS-Net, DeepSense, and ParallelCNN when $P_{f}=0.1$. (**b**) $P_{f}$ versus SNR (dB) of BSWSS-Net, CNNWSS-Net, DeepSense, and ParallelCNN when $P_{d}=0.9$.

**Figure 18:**
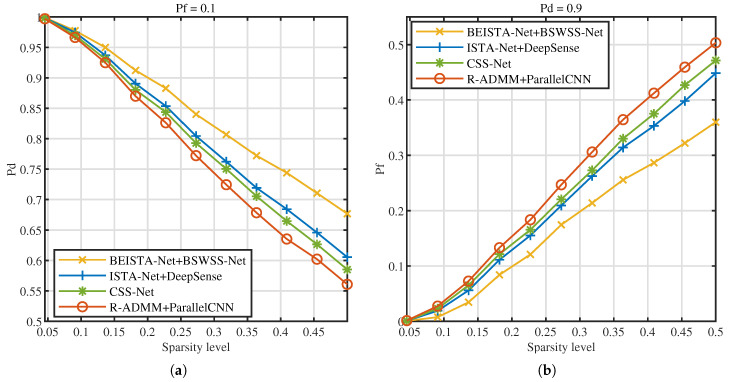
NR dataset with SNR ∈[−10,10] dB, P=8, L=16. (**a**) $P_{d}$ versus SNR (dB) of BEISTA-Net+BSWSS-Net, ISTA-Net+DeepSense, CSS-Net, and R-ADMM+ParallelCNN when $P_{f}\;=\;0.1$. (**b**) $P_{f}$ versus SNR (dB) of BEISTA-Net+BSWSS-Net, ISTA-Net+DeepSense, CSS-Net, and R-ADMM+ParallelCNN when $P_{d}=0.9$.

**Figure 19:**
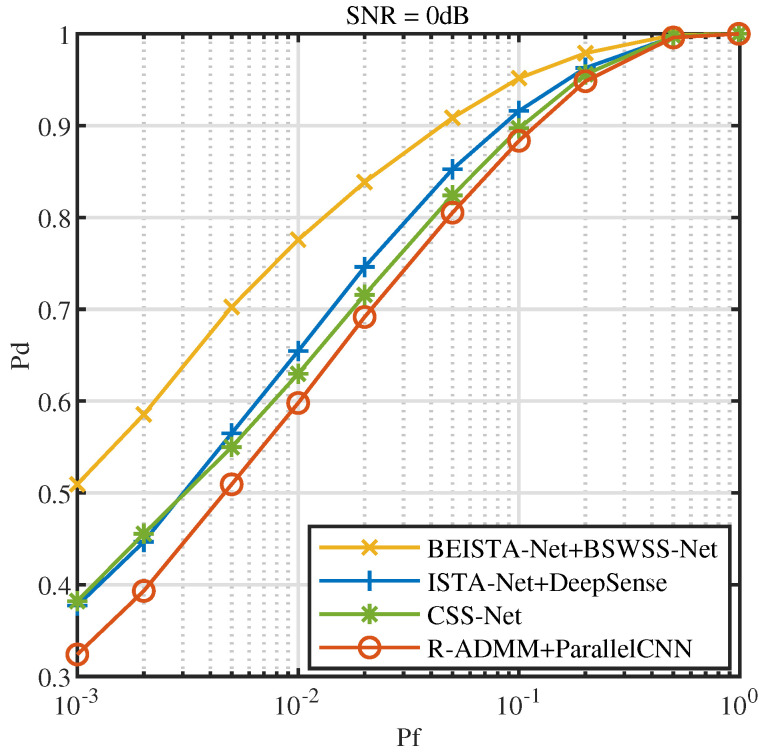
ROC curves when SNR=0dB in NR dataset, $\mu_{NR},\in \left(0,0.5\right]$, P=8, and L=16.

Then, we evaluate the performance in the NR dataset, with $\mu_{NR}\in \left(0,0.5\right]$, P=8, and L=16. We also study the effect of SNR and signal sparsity level on the CSS algorithm, as well as ROC. From Figure 17, Figure 18 and Figure 19, it is clear that the BEISTA-Net+BSWSS-Net joint algorithm outperforms the other three algorithms. Compared to the results in the TVWS dataset, the four algorithms show better performance in the NR dataset. This is because the label dimension in the NR dataset is smaller, leading to better classification results. In the NR dataset, BSWSS-Net outperforms the other three algorithms to a greater extent than in the TVWS dataset. Compared to the current state-of-the-art algorithms, the detection probability has been improved by nearly 4 percentage points when $P_{f}=0.1$ and SNR=0dB.

From the experimental results of the aforementioned two datasets, it is evident that the proposed BEISTA-Net+BSWSS-Net joint algorithm consistently achieves the highest detection probability and the lowest false alarm probability under varying noise conditions and sparsity levels. The algorithm is capable of adapting to scenarios with significant variations in channel conditions, achieving robust detection performance.

## 6. Conclusions

In this work, we propose a novel method for CSS that integrates BEISTA-Net and BSWSS-Net into a joint network. BEISTA-Net is employed for compressed signal reconstruction, while BSWSS-Net is employed for WSS. BEISTA-Net replaces the proximal mapping in ISTA with multiple convolution operations, incorporates the BSF extractor to obtain the BSF of wideband spectrum signals, and incorporates CA to enhance BSF during the iterative process. BSWSS-Net leverages neural networks to uncover hidden information in the frequency domain of wideband spectrum signals, thus obtaining channel state information. In evaluations across two datasets, the proposed method demonstrates robust performance across different levels of sparsity and SNR conditions compared to several baselines. However, the improvement in performance comes at the cost of increased computational complexity. While the proposed BEISTA-Net does not exhibit a significant change in running time compared to existing methods, it requires significantly higher FLOPs and video memory usage. This poses challenges for deployment on embedded devices. In other words, when actual hardware resources are limited, the real-time performance of the proposed method may be adversely affected. Future research can be advanced in three key directions: (1) further exploring the integration of traditional reconstruction algorithms with deep learning; (2) investigating the advanced application of deep attention mechanisms in CSS; and (3) reducing the complexity of the proposed CSS algorithm to lower deployment costs on practical hardware devices.

## Figures and Tables

**Table 1:** Hyperparameters of the BSWSS-Net.

	Layer	Filter	Stride	Padding
Convolutional Pooling Layer	$C_{1-1}$	4	2	1
	$C_{1-2}$	8	2	3
	$C_{1-3}$	12	2	5
	$C_{1-4}$	16	2	7
	$S_{1}$	4	4	0
	$C_{2}$	8	2	3
	$S_{2}$	4	4	0
	Layer		Out features	
Fully Connected Layer	$F_{1}$		256	
	$F_{2}$		$N_{c}$	

**Table 2:** Complexity analysis of different reconstruction algorithms.

Algorithm	# of FLOPs	# of Learnable Parameters	Execution Time (ms)	Video Memory Usage (MB)
ISTADR-Net	$2.6\times 10^{8}$	64	4.2	18.1
R-ADMM	$1.7\times 10^{8}$	$6.6\times 10^{4}$	11.4	14.8
ISTA-Net	$7.2\times 10^{8}$	$2.4\times 10^{6}$	2.1	49.4
BEISTA-Net	$7.3\times 10^{8}$	$2.5\times 10^{6}$	3.9	56.0

**Table 3:** Complexity analysis of different WSS algorithms.

Dataset	Algorithm	# of FLOPs	# Learnable Parameters	Execution Time (ms)	Video Memory Usage (MB)
NR dataset	DeepSense	$1.7\times 10^{6}$	$8.6\times 10^{4}$	0.39	1.44
	ParalellCNN	$1.2\times 10^{6}$	$4.3\times 10^{5}$	0.56	1.82
	CNNWSS-Net	$1.6\times 10^{6}$	$3.1\times 10^{5}$	0.50	1.40
	BSWSS-Net	$6.3\times 10^{5}$	$3.0\times 10^{5}$	0.38	1.28
TVWS dataset	DeepSense	$1.8\times 10^{6}$	$1.0\times 10^{5}$	0.40	1.59
	ParalellCNN	$1.1\times 10^{6}$	$4.4\times 10^{5}$	0.58	1.85
	CNNWSS-Net	$1.6\times 10^{6}$	$3.2\times 10^{5}$	0.52	1.43
	BSWSS-Net	$6.4\times 10^{5}$	$3.1\times 10^{5}$	0.38	1.30

## Data Availability

Data are contained within the article.
